# Alternative lengthening of telomeres: from molecular mechanisms to therapeutic outlooks

**DOI:** 10.1186/s13578-020-00391-6

**Published:** 2020-03-10

**Authors:** Jia-Min Zhang, Lee Zou

**Affiliations:** 1 Massachusetts General Hospital Cancer Center, Harvard Medical School, Charlestown, MA 02129 USA; 2 Department of Pathology, Massachusetts General Hospital, Harvard Medical School, Boston, MA 02114 USA

**Keywords:** Telomere, Telomere maintenance mechanism, Alternative lengthening of telomeres (ALT), APBs, Phase separation, ALT telomeric DNA synthesis, RAD52, BLM, FANCM, Clinical therapy

## Abstract

To escape replicative senescence, cancer cells have to overcome telomere attrition during DNA replication. Most of cancers rely on telomerase to extend and maintain telomeres, but 4–11% of cancers use a homologous recombination-based pathway called alternative lengthening of telomeres (ALT). ALT is prevalent in cancers from the mesenchymal origin and usually associates with poor clinical outcome. Given its critical role in protecting telomeres and genomic integrity in tumor cells, ALT is an Achilles heel of tumors and an attractive target for cancer therapy. Here, we review the recent progress in the mechanistic studies of ALT, and discuss the emerging therapeutic strategies to target ALT-positive cancers.

## Background

Telomeres, the ends of chromosomes, are protected by a slew of telomere-binding proteins, which prevent telomeres from being recognized as DNA double-strand breaks (DSBs) [1, 2]. During the division of somatic cells, the very end of telomeres cannot be replicated by the lagging strand of replication fork, leading to telomere shortening in each cell cycle [3]. If cells continue to proliferate, telomere attrition eventually triggers cellular senescence, a barrier of tumorigenesis [4]. To acquire replicative immortality, cancer cells must overcome telomere shortening [5]. While the majority of cancers accomplish this by activating telomerase [6–8], a significant fraction (4–11%) uses a telomerase-independent but homologous recombination-based pathway to extend and maintain telomeres [8–10]. This mechanism is known as alternative lengthening of telomeres (ALT) [10–13].

### Hallmarks of ALT

The telomerase-independent ALT pathway was first identified in the *S. cerevisiae* telomerase mutant, and subsequently characterized in human cancer cell lines and tumors [14–16]. Telomeric DNA synthesis in ALT^+^ cells involves both intra- and inter-telomeric recombination and replication [17, 18]. ALT^+^ cells display several hallmarks at telomeres, including (1) ALT-associated PML bodies (APBs) [19], (2) heterogeneous telomere length [15], (3) abundant extrachromosomal telomere repeat (ECTR) [20, 21], and (4) high levels of telomere sister chromatid exchange (T-SCE) [22]. Recent studies also found variants of telomeric repeats at telomeres and insertions of telomeric repeats across the genome in ALT^+^ cells [23, 24]. Using these ALT markers, a spectrum of ALT^+^ cancers are identified, revealing an association of ALT activation with cancers originated from the mesenchymal origin [9, 10, 25]. Sequencing of cancer genomes discovered that mutations of the ATRX/DAXX complex and the histone variant H3.3 are prevalent in ALT^+^ cancers [26–31]. Although significant progress has been made in phenotypic and sequencing analyses of ALT^+^ cells and cancers, ALT is still poorly understood as a pathway at the molecular level. The recent development of assays for telomeric DNA synthesis in ALT^+^ cells has finally allowed us to gain insights into the molecular mechanism of ALT. Here, we will review the recent findings on the molecular mechanisms of the ALT pathway and discuss the potential strategies to target the ALT pathway in cancer therapy.

### APBs and spatial regulation of ALT

APBs are unique nuclear structures containing the promyelocytic leukemia (PML) protein and telomeric DNA, and they are specifically present in ALT^+^ cells [19]. APBs typically contain clusters of multiple telomeres and numerous proteins involved in DNA repair, recombination and replication, making them an ideal environment for telomere recombination and DNA synthesis [19, 32–36]. Indeed, disruption of APBs by depleting PML not only abolishes telomere clustering, but also blocks ALT telomeric DNA synthesis in G2 cells [37, 38]. However, how APBs are assembled and how they promote ALT activation is still not fully understood.

**Fig. 1:**
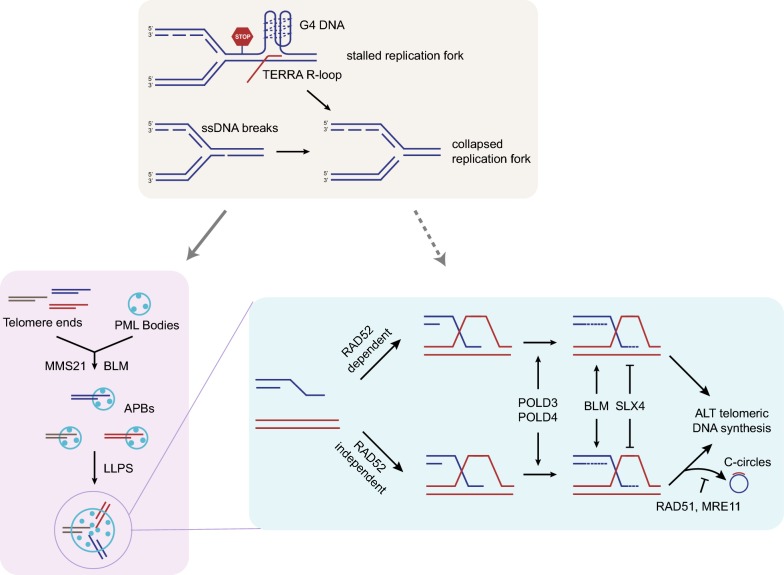
Framework of the ALT pathways. (Upper section) The replication stress at telomeres may be a trigger for ALT activation. The accumulation of R-loops, G-quadruplexes, and DNA single-strand breaks at telomeres may interfere with DNA replication, leading to collapse of replication forks and formation of one-ended DSBs. (Lower left section) the replication stress or DNA damage at telomeres may induce SUMOylation of telomere proteins, which recruit PML and trigger APB formation through SUMO/SIM-mediated LLPS. The clustering of telomeres and enrichment of DNA repair, recombination, and replication proteins in APBs may drive ALT efficiently. (Lower right section) In APBs, BIR is triggered by the one-ended DSBs at telomeres. ALT can take place through RAD52-dependent and -independent BIR pathways. The conservative DNA replication during BIR is dependent on POLD3/POLD4, promoted by BLM, and inhibited by SLX4. C-circles are generated by the RAD52-independent BIR pathway, which is suppressed by RAD51 and MRE11

PML bodies are membraneless organelles assembled through multivalent SUMO-SIM (SUMO-interacting motif) interactions among PML molecules, a process with features of liquid–liquid phase separation (LLPS) [39, 40]. A recent study proposed that APBs are also formed through LLPS (Fig. 1) [41]. In this study, fusion proteins containing multiple SUMO and SIMs were tethered to telomeres in ALT^–^ cells, resulting in PML-telomere colocalization and telomere clustering in nuclear condensates resembling APBs in ALT^+^ cells. Interestingly, although these poly-SUMO/SIM-induced condensates display features of LLPS, they are not sufficient for ALT DNA synthesis. Only when the BLM helicase was overexpressed in these ALT^–^ cells, several ALT-associated phenotypes, including heterogeneous telomere length, C-circles formation, and mitotic DNA synthesis (MiDAS) at telomeres, were detected [41]. It is possible that the enrichment of SUMO/SIM at telomeres recruits PML and triggers LLPS, driving multiple telomeres into condensates. However, clustering of telomeres does not appear to be sufficient to activate the ALT pathway. Overexpression of BLM apparently generates a signal critical for ALT activation in these telomere condensates. Interestingly, endogenous BLM is a component of APBs and critical for APB formation and ALT telomeric DNA synthesis [35, 38, 42]. While the induction of telomere LLPS and overexpression of BLM recapitulate important steps for ALT activation, how telomere LLPS and ALT activation are naturally triggered in ALT^+^ cells remains to be investigated.

The formation of APBs has been linked to the replication stress or DNA damage at telomeres. Loss of proteins that suppress replication stress or DNA damage, such as SMARCAL1, FANCD2, and FANCM, increases APB formation [43–46]. The replication stress at telomeres may lead to collapse of replication forks, which may promote APB formation through MMS21-mediated SUMOylation of shelterin and other telomere-binding proteins [47]. Interestingly, overexpression of BLM or RAD52 promotes APB formation, telomere clustering, and MiDAS at telomeres [41, 48], whereas overexpression of SLX4 reduces APBs and shortens telomere extension [48]. These results suggest that BLM and RAD52 may play opposing roles from SLX4 in regulating ALT telomeric DNA synthesis, and that ALT telomeric DNA synthesis may also affect the dynamics of APBs.

### Framework of the ALT pathway

The ALT pathway was initially characterized in the budding yeast mutant lacking functional telomerase [49]. In yeast, Rad51 is the key recombinase in the homologous recombination (HR) pathway [50]. Rad52 is critical for the binding of Rad51 to single-stranded DNA and also has the ability to anneal complementary ssDNA [51, 52]. There are two distinct types of ALT in yeast: Rad51- and Rad52-dependent type I survivors maintain telomeres by amplifying repetitive subtelomeric sequences, and Rad52-dependent but Rad51-independent type II survivors maintain telomeres by expanding the telomeric repeats [53]. The extension of telomeres by the ALT pathway in human cells is thought to be more similar to that in type II survivors [15, 54]. However, due to the lack of methods to directly monitor ALT DNA synthesis, little was known about how the ALT pathway operates in ALT^+^ human cells. Recently, the development of several assays to model or monitor ALT DNA synthesis, including break-induced telomere synthesis (BITS) [55, 56], MiDAS at telomeres [41, 57, 58], and ALT telomere synthesis in APBs (ATSA) [38], has allowed us to unravel the molecular mechanisms of ALT.

Break-induced replication (BIR) is a repair process initiated by one-ended DSBs at collapsed replication forks and extended by conservative DNA replication [59–61]. The yeast Pol32 and human POLD3/4 proteins, accessory subunits of the DNA polymerase, are important for BIR [56, 62, 63]. In ALT^+^ human cells, telomeres undergo conservative DNA replication in a POLD3/4-dependent manner, thereby linking ALT to BIR [64]. Human RAD52 is important for BIR at collapsed forks and MiDAS at fragile sites [65, 66]. Similar to that in the yeast type II survivors, the ALT pathway in human cells is dependent on RAD52 but not RAD51 [38, 41, 56, 57]. Telomeric DSBs generated by the TRF1-FOK1 nuclease fusion induce telomere clustering and BITS [55, 56]. Depletion of RAD51 impairs telomere clustering but does not affect BITS. Perhaps because ALT^+^ cells have high replication stress at telomeres, telomeric MiDAS is more robust in ALT^+^ cells than in ALT^–^ cells [57]. Telomeric MiDAS results in conservative DNA replication, recapitulating a phenotype of ALT^+^ cells. Depletion or inhibition of RAD52 decreases telomeric MiDAS. In contrast, RAD51 depletion increases telomeric MiDAS, fragile telomeres, and telomere dysfunction-induced foci (TIF) [57]. We recently showed that depletion of RAD52 decreases ATSA, the natural ALT telomere synthesis at APBs in G2 cells [38]. Depletion of RAD51 has no effect on ATSA but increases C-circles, a marker of ALT [38, 67]. In vitro, RAD52 but not RAD51 promotes D-loop formation on telomeric DNA in the presence of RPA [38]. Overexpression of RAD52 promotes telomere synthesis in G2 and mitotic cells [41]. All these results suggest that RAD52 but not RAD51 is important for ALT in human cells. Instead of participating in ALT directly, RAD51 may suppress the fragility of telomeres by protecting stalled replication forks. However, considering that RAD51 was shown to be required for telomere extension in cells overexpressing BLM [48], we cannot exclude a role for RAD51 in a context-specific ALT pathway.

It is important to emphasize that the ALT pathway in human cells is not a simple linear pathway. In our recent studies, we noticed that although ATSA is decreased in the newly generated RAD52 KO cells, significant levels of ATSA remain detectable in the absence of RAD52 [38]. Furthermore, C-circles levels are not altered in RAD52 knockdown cells and newly generated RAD52 KO cells, suggesting that there is a RAD52-independent ALT pathway, which is responsible for C-circles generation. During the passage of RAD52 KO cells, telomeres are progressively shortened and eventually stabilized. Interestingly, C-circle levels are elevated in extensively passaged RAD52 KO cells, indicating that the RAD52-independent pathway becomes more active when telomeres become shorter. Depletion of BLM, POLD3 and POLD4 decrease ATSA in extensively passaged RAD52 KO cells, showing that the RAD52-independent ALT pathway still depends on APBs and BIR for DNA synthesis. Based on these results, we proposed that ALT is a bifurcated pathway involving RAD52-dependent and RAD52-independent BIR, at least in G2 cells (Fig. 1) [38].

In human cells, the ALT pathway may alter according to the cell-cycle status of cells and the replication stress or DNA damage at telomeres. A low dose of aphidicolin induces RAD52-mediated telomeric MiDAS in not only ALT^+^ but also ALT^–^ cells [57, 58], suggesting that MiDAS is a general mechanism dealing with replication stress at telomeres. Depletion of SLX4 blocks telomeric MiDAS but increases APBs, C-circles and ALT telomere extension [48, 58]. It is possible that telomeric MiDAS only represents a sub-pathway of ALT in M phase. In contrast to telomeric MiDAS, ATSA detects telomeric DNA synthesis at APBs in G2 cells without exogenous DNA damage or replication inhibitor [38]. ATSA is only observed in ALT^+^ but not ALT^–^ cells. Both RAD52-dependent and -independent ALT pathways contribute to ATSA in G2, but telomeric MiDAS seems to be largely if not entirely RAD52-dependent. Telomeric DSBs generated by TRF1-FOK1 induce BITS in unsynchronized cells, with ALT^+^ cells displaying more DNA synthesis than ALT^–^ cells [56]. Interestingly, both RAD51 and RAD52 are dispensable for BITS, suggesting that BITS may resemble the RAD52-independent ALT pathway [68]. Recently, RAD51AP1 was shown to be required for TRF1-FOK1-induced telomere clustering and BITS [69]. Depletion of RAD51AP1 diminishes several ALT hallmarks, including APBs, T-SCE, ECTR, and the localization of RAD52 and POLD3 to telomeres. Moreover, knockout of RAD51AP1 leads to telomere shortening. In addition to DNA repair proteins, several nuclear receptors are also implicated in ALT. Recently, an ALT telomere maintenance pathway mediated by the COUP-TFII/TR4-FANCD2-MUS81-POLD3 axis was reported [70]. The nuclear receptor NR2C/F may recruit the NuRD–ZNF827 complex to telomeres and promote telomere recombination through chromatin remodeling [23, 24, 71]. Further studies are needed to understand how these factors function in RAD52-dependent and/or -independent pathways.

### Regulatory circuitries of ALT

The DNA replication stress at telomeres may be an important trigger of ALT activation (Fig. 1). The ATRX-DAXX complex was shown to deposit the histone variant H3.3 to telomeric regions of the genome [72–74]. Mutations in ATRX, DAXX, and H3.3 are prevalent in ALT^+^ cancers [26–28]. Expression of ATRX suppresses ALT markers including APBs and C-circles [75, 76], suggesting that ATRX is a repressor of ALT. Loss of ATRX results in dysregulation of the telomere non-coding RNA *TERRA* during the cell cycle and *TERRA* upregulation [77]. *TERRA* is known to form RNA:DNA hybrids at telomeres, inducing replication stress [78]. Loss of ATRX may also lead to accumulation of G-quadruplexes at ALT telomeres, which also impose replication stress [76, 79]. Some of the proteins bound to *TERRA* or RNA:DNA hybrids may act to reduce replication stress and suppress ALT. For example, *TERRA*-binding proteins SFPQ and NONO suppress telomeric RNA:DNA hybrids, and depletion of SFPQ increases APBs and telomere recombination in ALT^+^ cells [80].

Both intrinsic and extrinsic DNA replication stress promote ALT-associated events [57, 81, 82], suggesting that the proteins involved in the replication stress response are important for ALT suppression. For example, the ATP-dependent DNA-annealing helicase SMARCAL1 may counter replication stress by promoting reversal of stalled replication forks [83]. SMARCAL1 associates with ALT telomeres and suppresses ALT phenotypes [84]. RAD51 is also required for fork reversal, and MRE11 is involved in the nucleolytic processing of revered replication forks [85–87]. Loss of RAD51 or MRE11 increases C-circles, indicating that fork reversal and the subsequent processing may play an important role in suppressing C-circles formation [38, 81]. In addition, the fork protection complex (FPC) containing TIMELESS and TIPIN suppresses telomeric MiDAS [57].

Fanconi anemia (FA) proteins are also implicated in the suppression of ALT. FANCD2 suppresses BLM-dependent telomere extension in ALT^+^ cells [44]. Recently, FANCM was also found to suppress ALT [45, 46, 88]. Loss of FANCM in ALT^+^ cells enhances ALT phenotypes, including telomere clustering in APBs, C-circles formation, and telomeric DNA synthesis in G2 cells [45, 46]. In addition, depletion of FANCM leads to accumulation of phosphorylated RPA, single-stranded DNA, and foci of H2AX and 53BP1 at ALT telomeres, showing that hyperactive ALT is associated with high levels of DNA damage at telomeres. Importantly, FANCM is found to be critical for the viability of ALT^+^ cells but not ALT^–^ cells [45, 46]. The toxicity of FANCM depletion from ALT^+^ cells may be attributed to several functions of FANCM in ALT suppression. The function of FANCM in ALT suppression is dependent its ATPase/translocase activity. FANCM restricts *TERRA* levels and suppresses telomeric R-loops, possibly through its R-loop unwinding activity [46]. In addition, FANCM interacts with BLM and may antagonize the function of BLM in ALT [46]. Finally, FANCM may suppress ALT by reversing and remodeling stalled replication forks to counter replication stress at telomeres, which is similar to the role of SMARCAL1 [43, 89]

BLM and SLX4 appear to play opposing roles in ALT (Fig. 1). Overexpression of BLM induces telomere extension, APBs, and C-circles in ALT^+^ cells, whereas depletion of BLM causes opposite effects [35, 48]. When APBs are induced in ALT^–^ cells by tethering SUMO/SIM fusions to telomeres, BLM overexpression triggers a number of ALT phenotypes [41]. Notably, the helicase activity of BLM is essential for ALT telomeric DNA synthesis [41]. In contrast to BLM, overexpression of SLX4 suppresses telomere extension, APBs, and C-circles [48]. It is proposed that SLX4 and its associated nucleases suppress ALT DNA synthesis by resolving intermediates of telomere extension. Recently, the SLX4-interacing protein SLX4IP was also shown to suppress ALT [90]. Interestingly, loss of BLM suppresses the exacerbated ALT phenotypes in cells lacking SLX4 and SLX4IP [90], which is reminiscent of the suppression of hyperactive ALT in FANCM-depleted cells by BLM loss [46]. In both cases, BLM activity seems to contribute to the hyper ALT phenotypes and the associated genomic instability.

Although BLM is clearly critical for ALT, its function in ALT is still an enigma. BLM might promote ALT by enhancing DSB end resection or processing/modeling recombination intermediates. In FANCM-depleted ALT^+^ cells, BLM may cause toxicity by driving hyper resection [46]. When APBs are induced in ALT^–^ cells, BLM overexpression leads to accumulation of RPA at telomeres, consistent with a role of BLM in resection [41]. However, depletion of DNA2, the nuclease that functions with BLM in resection, fails to suppress ALT in cells lacking SLX4IP [90]. If BLM is important for processing/remodeling certain recombination intermediates during BIR, this function is likely antagonized by SLX4. The proper balance between BLM and SLX4 may be important for keeping ALT activity at a productive but tolerable level. While the hyperactivity of BLM may be a cause of ALT-associated genomic instability, it should be noted that BLM is required for APB formation and ALT DNA synthesis [38, 42]. Thus, loss of BLM is expected to block ALT and suppress the genomic instability associated with hyperactive ALT regardless of whether hyperactive BLM is the cause.

The Shelterin complex is critical for telomere maintenance regardless of the ALT status [91]. While some Shelterin proteins may be involved in ALT, it is generally difficult to separate their ALT functions from their roles in telomere protection. Nonetheless, some known functions of Shelterin proteins likely influence ALT activity. For example, loss of TRF1 increases telomere fragility, which may trigger ALT [92]. TRF2 is required for the induction of R-loops at telomeres after oxidative DNA damage, and it may also promote ALT by increasing telomeric R-loops [93]. Both TRF1 and TRF2 are SUMOylated by MMS21, allowing them to localize to APBs [94]. TRF1 may contribute to APB formation through its interaction with PMLIV [95]. Recently, it was shown that SUMOylation of TRF2 promotes telomere clustering, APB formation and Telomeric MiDAS [41].

### Therapeutic outlooks

Although ALT is only active in about 4–11% of human cancers, it is prevalent in specific cancers, such as osteosarcoma, leiomyosarcoma, liposarcoma, glioblastoma, and neuroendocrine pancreatic cancer [9, 25, 26]. Moreover, the specific activation of ALT in tumors makes it a potential target for therapy. With the recent insights into the ALT pathway, several strategies have been proposed to exploit the dependency of tumors on ALT.

Since telomere length is heterogeneous in ALT^+^ cells, inhibition of ALT may lead to shortening of already short telomeres, causing loss of telomeres, toxic chromosome fusions, and cell death. Because APBs are important for ALT DNA synthesis [19, 38], disrupting APBs may abolish ALT activity in ALT^+^ cells. Indeed, disruption of APBs by PML knockdown results in telomere shortening in ALT^+^ cells [37]. Similarly, knockdown of MMS21, a SUMO ligase required for APB formation, also causes telomere shortening in ALT^+^ cells [47]. While blocking the ALT pathway may induce cell death in some ALT^+^ cells, whether this strategy can rapidly eliminate populations of ALT^+^ cells in tumors is still unclear. ALT^+^ cell populations seem to undergo progressive telomere shortening in the absence of ALT activity, and eventually enter senescence. Thus, blocking ALT in ALT^+^ tumor cells may reduce their oncogenic potential, but may not eliminate them in a timely fashion.

A second strategy to kill ALT^+^ cells is to induce ALT-specific synthetic lethality. ALT is a mechanism to extend short telomeres. If ALT^+^ tumor cells accumulate high levels of DSBs at telomeres, they will be increasingly dependent on ALT activity to extend broken telomeres and survive. Inhibition of the ATR kinase, a master regulator of the replication stress response, increases telomere fragility [96, 97]. This effect of ATR inhibition may be attributed to both the increase of replication stress at telomeres and the reduction in replication stress response. Furthermore, ATR is required for telomeric MiDAS in ALT^+^ cells [57], suggesting that it may contribute to ALT activity. Thus, inhibition of ATR in ALT^+^ cells not only induces DSBs at telomeres, but also prevents extension of broken telomeres through ALT, thereby creating a lethal situation in ALT^+^ cells. Given the promising effects of ATR inhibitors on some ALT^+^ cell lines [77], it would be important to test whether ATR inhibitors can effectively eliminate ALT^+^ tumors in mouse models and patients.

As mentioned above, hyperactive ALT is associated with high levels of DNA damage at telomeres and may cause cell death. Therefore, strategies that induce hyperactive ALT may also selectively kill ALT^+^ cells. For example, compounds that stabilize G-quadruplexes at telomeres may promote hyper activation of ALT, leading to death of ALT^+^ cells [98]. Because loss of FANCM results in hyperactive ALT and death of ALT^+^ cells, inhibitors of FANCM could potentially eliminate ALT^+^ tumors [45, 46]. As a proof of principle, an ectopic MM2 peptide disrupting the interaction between FANCM and BLM is sufficient to decrease the survival of ALT^+^ cells but has no effect on ALT^–^ cells [45]. Similarly, the small molecule inhibitor PIP-199 disrupts the FANCM-BLM interaction and suppresses the growth of ALT^+^ cancer cells.

Because ATRX is often lost in ALT^+^ cells, the absence of ATRX in ALT^+^ cells may provide a therapeutic opportunity. ATRX is involved in the restart of stalled replication forks [99], and in DNA repair synthesis and sister chromatid exchange during HR [100]. Loss of ATRX was shown to sensitize cells to DNA-damaging agents [101]. ATRX deficiency was also shown to promote accumulation of G-quadruplexes, making cells sensitive to a G4-stabilizing compound [102]. Furthermore, a mutant herpes simplex virus type 1 (HSV-1) infects ATRX-deficient cells much more efficiently than ATRX-proficient cells, selectively killing ATRX-deficient cells [103]. Thus, although strategies that selectively kill ATRX-deficient cells may not exploit the ALT pathway directly, they may eliminate the ATRX-deficient ALT^+^ tumors.

Targeting the ALT-specific telomere proteins may be another way to kill ALT^+^ cells. TSPYL5 is a protein that is specifically expressed in ALT^+^ cancer cells [104]. Interestingly, depletion of TSPYL5 induces USP7-dependent proteasomal degradation of POT1, a component of the shelterin complex, leading to death of ALT^+^ cells. Furthermore, knockdown of PML prevents POT1 degradation in ALT^+^ cells lacking TSPYL5, suggesting that POT1 becomes reliant on TSPYL5 in APBs. This finding provides an example of how ALT and APB can change the behaviors of telomere binding proteins, suggesting that targeting ALT-specific telomere proteins like TSPYL5 may induce genomic instability specifically at ALT telomeres, killing ALT^+^ cells selectively.

## Conclusions

**Fig. 2:**
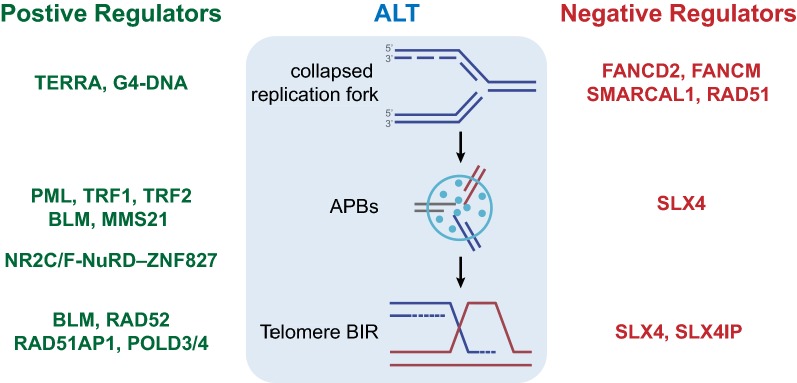
Positive and negative regulators of ALT. (Left) Positive regulators of ALT discussed in this article. (Middle) Major events that occur during the process of ALT. (Right) Negative regulators of ALT discussed in this article

In this review, we attempted to summarize the recent studies on the molecular basis of the ALT pathways. Collectively, these studies suggest that ALT is a complex BIR pathway initiated by the replication stress or DNA damage at telomeres. Although several key events for ALT activation are uncovered, how this pathway is specifically activated in ALT^+^ cells still requires further investigations. ALT activity can be detected at APBs in G2 cells, and even in mitotic cells. A number of positive and negative regulators of ALT have been identified (Fig. 2), but how they function together is still not fully understood. It is important to note that many of the current approaches to study ALT still rely on exogenous DNA damage or replication inhibitors. How the natural ALT activity in ALT^+^ cells is regulated remains an outstanding question. With a better understanding of the ALT pathway, we expect that more therapeutic strategies will be developed to exploit the ALT-associated vulnerabilities of ALT^+^ tumors. The next decade will be an exciting time for both basic and translational research of ALT.

## Abbreviations

ALT: Alternative lengthening of telomeres
APBs: ALT-associated PML bodies
ATSA: ALT telomere synthesis in APBs
BIR: Break-induced replication
BITS: Break-induced telomere synthesis
DSBs: DNA double-strand breaks
ECTR: Extrachromosomal telomere repeat
HR: Homologous recombination
LLPS: Liquid–liquid phase separation
MiDAS: Mitotic DNA synthesis
PML: Promyelocytic leukemia
T-SCE: Telomere sister chromatid exchange
